# Safety and efficacy of stent-assisted coiling with the pEGASUS-HPC stent in wide-necked intracranial aneurysms: a multicenter retrospective analysis

**DOI:** 10.1136/jnis-2025-023946

**Published:** 2025-09-24

**Authors:** Mohammad Almohammad, Ali Khanafer, Mete Dadak, Christopher Nimsky, Alexander Grote, Bayan Alhaj Moustafa, Islam El Malky, Lisa Hekers, Abdallah Aburub, Zakarya Ali, Mariana Gurschi, Julia Korthäuer, Stephan Felber, Hans Henkes, André Kemmling

**Affiliations:** 1 Fachbereich Medizin, Philipps-Universität Marburg, Marburg, Germany; 2 Department of Neuroradiology, University Hospital of Giessen and Marburg Campus Marburg, Marburg, Germany; 3 Neuroradiology, Klinikum Stuttgart Katharinenhospital, Stuttgart, Germany; 4 Department of Radiology, St Vincenz Hospital Paderborn, Paderborn, Germany; 5 Department of Neurosurgery, University Hospital of Giessen and Marburg Campus, Marburg, Germany; 6 Neurology, South Valley University, Qena, Qena Governorate, Egypt; 7 Central Institute for Diagnostic and Interventional Radiology Neuroradiology and Nuclear Medicine, Offenbach am Main, Germany; 8 Institute for Diagnostic and Interventional Radiology and Neuroradiology, Koblenz, Germany; 9 Klinik für Neuroradiologie, Klinikum Stuttgart, Stuttgart, Germany; 10 Department of Neuroradiology, Rhön Klinikum, Campus Bad Neustadt, Bad Neustadt an der Saale, Germany

**Keywords:** Aneurysm, Coil, Stent, Intervention

## Abstract

**ABSTRACT:**

**Background:**

Stent-assisted coiling is widely used for the treatment of wide-necked intracranial aneurysms. The pEGASUS-HPC stent features a hydrophilic polymer coating designed to reduce thrombogenicity, potentially improving safety, particularly in ruptured aneurysms and complex configurations.

**Objective:**

To evaluate the safety, efficacy, and technical feasibility of stent-assisted coiling using the pEGASUS-HPC stent in the treatment of wide-necked intracranial aneurysms.

**Methods:**

We retrospectively analyzed 223 patients with 251 wide-necked aneurysms treated at six centers between July 2021 and March 2025. Primary endpoints included angiographic occlusion (modified Raymond–Roy classification, MRRC), periprocedural complications, and clinical outcome (modified Rankin Scale, mRS) at discharge and follow-up. Subgroup analysis was performed for ruptured aneurysms and bifurcation cases requiring Y-stenting.

**Results:**

Stent deployment and coiling were technically successful in all cases. Immediate complete occlusion (MRRC I) was achieved in 92.4% of aneurysms, increasing to 96.8% at follow-up. The overall retreatment rate was 2.2%. Periprocedural complications occurred in 1.6% of cases, consisting of two minor perforations in unruptured aneurysms, both managed without clinical consequences. In ruptured cases a favorable outcome (mRS ≤2) was observed in 71.1%, with mortality confined to Hunt and Hess grade V or basilar artery aneurysms. Y-stenting was performed in 15.9% of cases without technical failure. No perforations occurred in ruptured cases.

**Conclusion:**

Stent-assisted coiling with the pEGASUS-HPC stent appears technically feasible and effective for wide-necked aneurysms, including acute ruptured cases and those requiring Y-stenting, with high occlusion rates and low complication rates.

**WHAT IS ALREADY KNOWN ON THIS TOPIC**

- Stent-assisted coiling is commonly used for treating wide-necked intracranial aneurysms, but thromboembolic complications remain a concern. Coated stents such as the pEGASUS-HPC have shown promising safety and deliverability in small series, yet robust multicenter data have been lacking.

**WHAT THIS STUDY ADDS**

- This large multicenter study demonstrates high occlusion rates and a low complication rate with the pEGASUS-HPC stent, including in ruptured aneurysms and complex bifurcation anatomies requiring Y-stenting. The findings suggest that the antithrombogenic coating may reduce thromboembolic risks in both elective and acute settings.

**HOW THIS STUDY MIGHT AFFECT RESEARCH, PRACTICE, OR POLICY**

- These results support the expanded use of coated stents in neurointerventional practice and provide a basis for future prospective studies comparing coated and uncoated stent systems. They may also inform antiplatelet management strategies, particularly in the setting of acute subarachnoid hemorrhage.

## Introduction

 Intracranial aneurysms are focal dilations of cerebral arteries with a high risk of rupture, potentially leading to subarachnoid hemorrhage (SAH) associated with significant morbidity and mortality. Endovascular coiling has emerged as a key minimally invasive alternative to surgical clipping, especially after the International Subarachnoid Aneurysm Trial (ISAT) which demonstrated favorable outcomes in selected patients.^1^,^4^ Wide-necked aneurysms present a particular challenge due to an increased risk of coil prolapse and often require adjunctive techniques for stable occlusion. Stent-assisted coiling (SAC) is frequently used in these scenarios to provide a scaffold across the aneurysm neck and promote endothelialization.^5 6^ Certain conditions limit the use of SAC—for example, in acutely ruptured aneurysms, SAC must be applied with caution due to the need for antiplatelet therapy, which may increase the risk of hemorrhagic complications during the acute phase of SAH.^7 8^

Y-stenting has become a widely accepted technique for treating bifurcation aneurysms when a single stent cannot adequately cover the aneurysm neck. In such cases, a Y-shaped stent configuration facilitates safe and effective coil placement across both branches.^9^

The pEGASUS-HPC stent (Phenox GmbH, Bochum, Germany) is a low-profile, self-expanding, open-cell, laser-cut device designed for endovascular treatment of wide-necked aneurysms, dissections, and intracranial stenoses.^10 11^ It can be delivered through standard 0.0165 inch coiling microcatheters without exchange and features a hydrophilic polymer coating (HPC) that reduces platelet adhesion and thrombogenicity. This may be especially advantageous in the setting of acute SAH, where minimizing dual antiplatelet therapy is critical.^12 13^

Early case series have reported high technical success and favorable safety with the pEGASUS-HPC stent.^10 14^ However, broader clinical experience remains limited and robust multicenter data are lacking. In daily practice, other commonly used uncoated stents include the Neuroform Atlas, LVIS Jr, Enterprise, and Solitaire systems, which have demonstrated variable occlusion and complication rates but lack antithrombogenic surface modification.^15^,^17^

This is the first study to systematically evaluate the pEGASUS-HPC stent in a large multicenter cohort including a substantial number of complex bifurcation aneurysms treated with Y-stenting. By covering both ruptured and unruptured aneurysms in diverse anatomical locations, our study provides real-world insights into the safety, efficacy, and technical performance of the device, helping to define its role in contemporary neuroendovascular therapy.

## Methods

### Study design

This retrospective multicenter study included consecutive patients treated between June 2021 and March 2025 across six neurovascular centers.

### Study population

All patients with wide-necked intracranial aneurysms treated with SAC using the pEGASUS-HPC stent were included. Wide-necked aneurysms were defined as having a neck diameter of ≥4 mm or a dome-to-neck ratio of <2, as defined in previous studies.^18^ Both elective (unruptured) and acute (ruptured) cases were eligible. No cases were excluded from the analysis.

In cases of bifurcation aneurysms with unfavorable branch angulation or wide-neck morphology that precluded sufficient coverage with a single stent, Y-stenting was performed to achieve stable neck reconstruction.

### Study procedures

**Figure 1:**
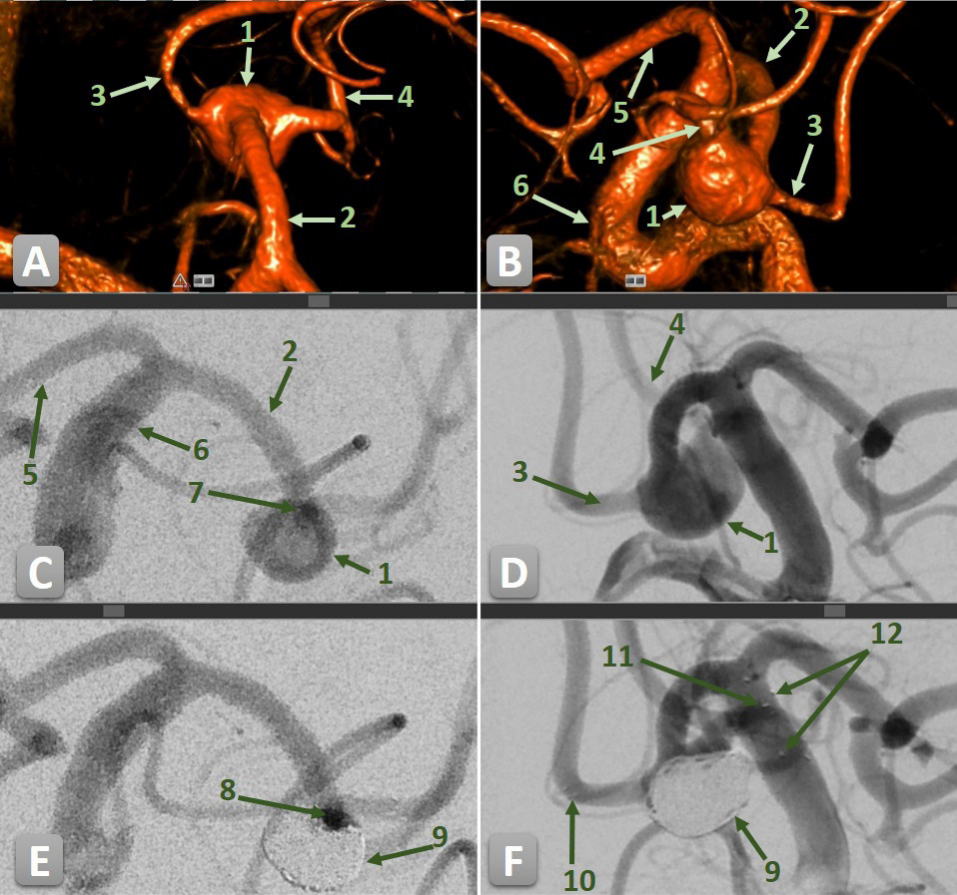
DSA of a wide-necked unruptured aneurysm at the bifurcation of the middle cerebral artery (MCA) treated with stent-assisted coiling (SAC) using two pEGASUS-HPC stents in a Y-configuration. (1) MCA aneurysm. (2) M1 segment of the MCA. (3) Posterior M2 branch. (4) Anterior M2 branch. (5) Anterior cerebral artery. (6) Internal carotid artery. (7) Overlapping origin of both M2 branches before SAC. (8) Patent M2 branches after SAC. (9) Coiled aneurysm. (10) Distal markers of the anterior pEGASUS-HPC stent. (11) Distal markers of the posterior pEGASUS-HPC stent. (12) Proximal markers of both stents. (**A, B**) 3D rotational angiograms showing the vascular anatomy prior to SAC. (**C, D**) Pre-treatment 2D angiograms showing the aneurysm and bifurcation anatomy. (**E, F**) Post-treatment 2D angiograms following deployment of both pEGASUS-HPC stents in Y-configuration and coil embolization, with visible proximal and distal stent markers.

All procedures were performed under general anesthesia using biplane DSA and 3D rotational angiography to assess aneurysm morphology and parent vessel anatomy (see figures1  2). A triaxial access system was used in all cases. Depending on vascular anatomy and operator preference, either transfemoral or transradial access was employed. An Excelsior SL-10 microcatheter (0.0165 inch × 150 cm; Stryker) was used for both stent deployment and coil embolization.^19^ At the beginning of each procedure all patients received an IV bolus of 4000 IU heparin.

**Figure 2:**
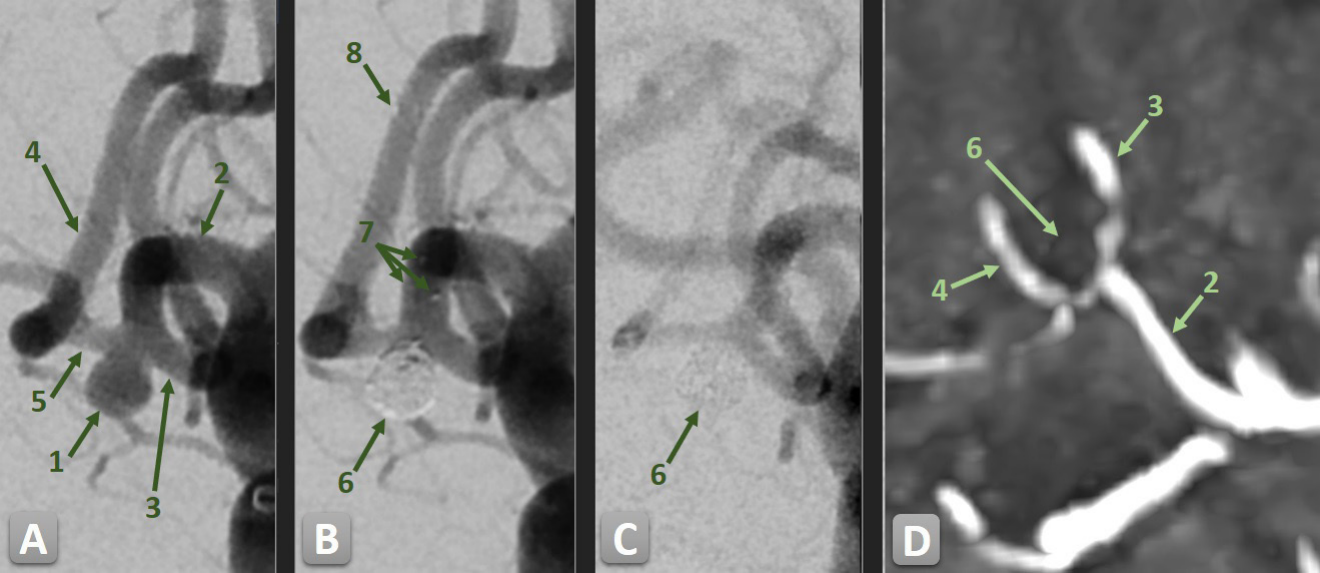
DSA of a relatively wide-necked unruptured aneurysm of the anterior communicating artery (AComA) treated with stent-assisted coiling (SAC) using a single pEGASUS-HPC stent, providing sufficient neck coverage. (1) AComA aneurysm. (2) A1 segment of the left anterior cerebral artery (ACA). (3) A2 segment of the left ACA. (4) A2 segment of the right ACA. (5) AComA. (6) Coiled aneurysm. (7) Proximal stent markers in the A1 segment of the left ACA. (8) Distal stent markers in the A2 segment of the right ACA. (A) Pre-treatment 2D angiogram showing the aneurysm and adjacent vascular anatomy. (B) Post-treatment 2D angiogram following stent deployment and coil embolization showing proximal and distal stent markers. A small residual perfusion at the aneurysm neck is visible. (C) Angiographic follow-up at 3 months showing progressive, now complete occlusion of the aneurysm sac. The weak contrast opacification is due to the proximal catheter position. (D) Last MRI follow-up (time-of-flight) at 2 years confirming persistent and complete aneurysm occlusion.

Antiplatelet therapy for unruptured aneurysms varied slightly between participating centers and followed two institutional protocols. Five centers used Protocol A, in which patients received acetylsalicylic acid (ASA) 100 mg and prasugrel 10 mg daily for at least 5 days prior to the intervention. One center followed Protocol B, where patients received ASA 100 mg daily for at least 5 days followed by a 180 mg ticagrelor loading dose 2 hours before the procedure and then continued with ticagrelor 90 mg twice daily thereafter.

In Protocol A, four of the five centers continued dual antiplatelet therapy (DAPT) for 3 months, while one center applied 6 months of DAPT. In Protocol B, DAPT was uniformly continued for 3 months. Thereafter, all centers transitioned patients to single antiplatelet therapy with ASA 100 mg daily as lifelong secondary prophylaxis.

For ruptured aneurysms, platelet inhibition was performed according to a standardized protocol. A weight-adjusted IV bolus of tirofiban was administered during stent deployment, in accordance with a previously published protocol.^8^ Coil embolization was performed via the same microcatheter. A final angiographic run was conducted to confirm complete aneurysm occlusion and to exclude procedure-related complications. Immediately after the procedure, and after ruling out complications, a loading dose of 60 mg prasugrel was administered. Starting the following day, patients received prasugrel 10 mg daily for 3–6 months, after which they were switched to ASA 100 mg daily for long-term secondary prevention.

Platelet inhibition was monitored in all protocols using the VerifyNow system (Werfen) and antiplatelet dosages were adjusted accordingly.

### Clinical endpoints

Clinical outcomes were assessed using the modified Rankin Scale (mRS) at discharge and during follow-up visits at 3 months, 6 months, and 1 year. Procedural safety was evaluated by documenting any procedure-related complications including acute vessel occlusion, in-stent thrombosis, intraoperative aneurysm rupture, and progression of SAH in ruptured aneurysms.

### Imaging endpoints

The primary imaging outcome was the degree of aneurysm occlusion following SAC with the pEGASUS-HPC device. Immediate procedural success was assessed using the modified Raymond–Roy classification (MRRC) based on angiographic findings obtained directly after treatment. A follow-up DSA was performed at either 3 or 6 months, according to institutional protocols, to evaluate aneurysm status. Long-term occlusion and vessel patency were subsequently assessed on an annual basis using either time-of-flight (TOF) magnetic resonance angiography (MRA) or CT angiography (CTA).

### Data collection

Demographic, clinical, imaging, procedural, and follow-up data were retrospectively collected from patient records and systematically organized using Microsoft Excel. This included baseline characteristics, aneurysm morphology, treatment strategies, and clinical outcomes. Detailed procedural information such as access route, devices used, techniques applied, and intra- or periprocedural complications was meticulously documented. Follow-up imaging studies were evaluated using the institutional Picture Archiving and Communication System (PACS) by experienced neuroradiologists. To ensure data integrity and consistency across centers, standardized documentation templates were used and all entries were subject to internal cross-checks and plausibility validation.

### Statistical analysis

Continuous variables were reported as mean±SD or median (IQR), depending on data distribution. Categorical variables were summarized as counts and percentages. Group comparisons were performed using t-tests or Mann–Whitney U tests for continuous data and χ^2^ or Fisher’s exact tests for categorical variables. Functional outcomes (mRS) at discharge and final follow-up were compared using the Wilcoxon signed-rank test. Results were expressed as medians with interquartile ranges (IQRs). Statistical significance was defined as P<0.05. All analyses were conducted on complete cases using Jamovi version 27.0 (IBM Corp, Armonk, New York, USA).

## Results

### Demographic and clinical characteristics

The study included 223 patients with a total of 251 wide-necked intracranial aneurysms. The mean±SD age was 63.6±10.7 years and 133 patients (59.6%) were female. Aneurysms were predominantly located in the anterior circulation (171 aneurysms, 68.1%), with the anterior communicating artery (AComA) being the most common site (89 aneurysms, 35.5%) followed by the basilar artery (BA) in 69 cases (27.5%) and the middle cerebral artery (MCA) in 51 (20.3%). Midline aneurysms involving the BA or AComA accounted for 158 cases (63.0%), whereas lateralized aneurysms were found on the left in 59 cases (23.5%) and on the right in 34 cases (13.5%). A total of 24 aneurysms (9.6%) were already ruptured at presentation while 227 (90.4%) were unruptured. The mean±SD neck width was 4.9±1.9 mm, the mean±SD sac width was 5.9±3.1 mm, and the mean±SD sac depth was 5.2±3.1 mm, resulting in an average dome-to-neck ratio of 1.25±0.7. Baseline characteristics are summarized in table 1.

**Table 1:** Demographic, clinical, and radiological characteristics of patients with intracranial aneurysms

Parameters	
Age, years, mean±SD	63.6±10.7
Female, n (%)	133 (59.6%)
Aneurysm location, n (%)	
AComA	89 (35.5%)
BA	69 (27.5%)
MCA	51 (20.3%)
PComA	19 (7.6%)
ICA	21 (8.4%)
PICA	2 (0.8%)
Anterior vs posterior	
Anterior	171 (68.1%)
Aneurysm laterality	
Left	59 (23.5%)
Right	34 (13.5%)
Midline (BA or AcomA)	158 (63.0%)
Aneurysm neck width, mm	4.9±1.9
Aneurysm sac width, mm	5.9±3.1
Aneurysm sac depth, mm	5.2±3.1
Dome-to-neck ratio	1.25±0.7
Ruptured aneurysms	24 (9.6%)
Non-ruptured aneurysms	227 (90.4%)
In ruptured aneurysms (n=24, 9.6%):	
Hunt and Hess grade	
I	1 (4.2%)
II	3 (12.5%)
III	6 (25%)
IV	4 (16.7%)
V	5 (20.8%)
Fisher grade	
I	7 (29.2%)
II	4 (16.7%)
III	5 (20.8%)
IV	8 (33.3%)

AComA, anterior communicating artery; BA, basilar artery; ICA, internal carotid artery; MCA, middle cerebral artery; PComA, posterior communicating artery; PICA, posterior inferior cerebellar artery.

### Clinical endpoints

In patients with unruptured aneurysms the median mRS at discharge was 0 (0–0), indicating an absence of neurological deficits in nearly all cases. This favorable outcome persisted through the last follow-up, with a median mRS of 0 (0–0). The average duration of hospitalization was 2.4±0.8 days.

In patients with ruptured aneurysms the median mRS at discharge was 3 (2–5), reflecting moderate to moderately severe disability in the acute phase. Functional outcomes improved markedly during early recovery, with a median mRS of 0 (0–2) at 3 months and a narrower IQR of 0–1 at the last documented follow-up.

The median (IQR) time from aneurysm rupture to endovascular treatment was 0 (0–1) days, indicating early intervention in the majority of ruptured cases. The mean length of hospitalization in ruptured cases was 16±9.8 days (see table 2).

**Table 2:** Procedural, device, and vessel characteristics, radiographic and clinical outcomes

Parameters	
Number of devices	
1	201 (84.1%)
2 (Y-stenting)	40 (15.9%)
Stent diameter, mm, mean±SD	3.7±0.5
Stent length, mm, mean±SD	21.6±3.8
Second device diameter (in Y-stenting cases), mm, mean±SD	3.8±0.5
Second device length (in Y-stenting cases), mm, mean±SD	23.5±3.4
Number of used coils, median (IQR)	5 (1–13)
Proximal artery diameter, mm, mean±SD	2.9±1.2
Distal artery diameter, mm, mean±SD	1.8±0.6
Proximal second artery diameter (in Y-stenting cases), mm, mean±SD	2.6±0.8
Distal second artery diameter (in Y-stenting cases), mm, mean±SD	1.5±0.7
EVD insertion in already ruptured cases (n=24)	16 (66.7%)
Days from aneurysm rupture to intervention, median (IQR)	0 (0–1)
Days of hospitalization in unruptured aneurysms, mean±SD	2.4±0.8
Days of hospitalization in ruptured aneurysms, mean±SD	16±9.8
MRRC post-intervention	
Class I	232 (92.4%)
Class II	6 (2.4%)
Class IIIa	3 (1.2%)
Class IIIb	10 (4.0%)
MRRS at 3 or 6 months follow-up	
Class I	243 (96.8%)
Class II	5 (2%)
Class IIIa	0 (0%)
Class IIIb	3 (1.2%)
mRS in unruptured aneurysms at	
Discharge	0 (0–0)
3 or 6 months follow-up	0 (0–0)
Last follow-up	0 (0–0)
mRS in ruptured aneurysms at	
Discharge	3 (2–5)
3 or 6 months follow-up	0 (0–2)
Last follow-up	0 (0–1)

EVD, external ventricular drain; MRRC, modified Raymond–Roy Occlusion Classification; mRS, modified Rankin Scale.

### Imaging and procedural endpoints

Immediate post-procedural angiographic results showed complete aneurysm occlusion (MRRC Class I) in 232 aneurysms (92.4%) (see figures1  2). Residual neck filling (Class II) was observed in six patients (2.4%) while residual aneurysm filling was noted in three aneurysms (1.2%) as Class IIIa and in 10 aneurysms (4.0%) as Class IIIb.

At follow-up (3 or 6 months), complete occlusion (MRRC Class I) was confirmed in 243 aneurysms (96.8%). Residual neck filling (Class II) persisted in five aneurysms (2%) and residual aneurysm filling (Class IIIb) was seen in three aneurysms (1.2%). These cases underwent retreatment which resulted in complete occlusion (MRRC Class I) in all.

A median of five coils (IQR 1–13) was used to achieve stable aneurysm occlusion, depending on individual aneurysm morphology. Technical success of device deployment was achieved in all cases. A single pEGASUS-HPC stent was implanted in 201 patients (84.1%), while 40 patients (15.9%) underwent dual stent implantation using a Y-configuration. The mean diameter and length of the primary stent were 3.7±0.5 mm and 21.6±3.8 mm, respectively. In Y-stent procedures, the second stent had a mean diameter of 3.8±0.5 mm and a mean length of 23.5±3.4 mm (table 2). No deaths occurred among patients with unruptured aneurysms.

In the ruptured subgroup all deceased patients (3/3) presented with Hunt and Hess grade V at admission and had basilar artery aneurysms. A statistically significant association of these two factors with mortality was observed in this subgroup (P=0.0049). Other factors such as age, gender, stenting technique, and treatment delay showed no significant correlation with fatal outcome.

### Observed complications

Complications occurred in four patients (1.6%), two cases (0.8%) in the ruptured aneurysm group and two (0.8%) in the unruptured group. The remaining 247 patients (98.4%) experienced no procedural or post-procedural adverse events. Two perforations were observed, both in unruptured aneurysms, one caused by the microwire and the other by the first deployed coil. Both were successfully treated with immediate coiling and resulted in no clinical deterioration.

Among the ruptured aneurysms, two complications were reported: one case (0.4%) of transient in-stent thrombosis that resolved spontaneously following administration of a tirofiban bolus and one case (0.4%) of SAH progression observed on follow-up imaging. Notably, none of the complications required retreatment.

## Discussion

This multicenter retrospective study presents detailed clinical and radiographic outcomes of 251 wide-necked intracranial aneurysms treated with the pEGASUS-HPC stent. The results show high rates of both immediate and durable aneurysm occlusion, low periprocedural complication rates, and favorable functional outcomes. Notably, the stent proved effective even in complex cases, including acutely ruptured aneurysms and bifurcation aneurysms requiring Y-stenting due to inadequate neck coverage with a single stent. These findings support the pEGASUS-HPC stent as a safe and effective device for SAC in both elective and emergent neuroendovascular interventions.

### Efficacy in aneurysm occlusion

In our cohort the pEGASUS-HPC stent achieved an immediate complete occlusion rate (MRRC Class I) of 92.4%, indicating a high level of technical and therapeutic efficacy. Follow-up angiography demonstrated sustained aneurysm exclusion in 96.8% of cases, confirming the long-term durability of the treatment. The retreatment rate was low and, in cases with initial incomplete occlusion, successful re-embolization was achieved without technical complications. Notably, the design of the stent enabled safe and reliable microcatheterization through the struts when necessary, facilitating secondary interventions and underscoring its procedural flexibility in complex anatomies.

### Safety profile and complications

The overall periprocedural complication rate in our study was 1.6%, underscoring the favorable safety profile of the pEGASUS-HPC stent. Importantly, no permanent morbidity or mortality occurred among patients with unruptured aneurysms and none of the observed complications required retreatment. This low rate of adverse events is particularly notable given the inclusion of acutely ruptured aneurysms (9.6%) and complex bifurcation anatomies treated with Y-stenting (15.9%). In patients with ruptured aneurysms, early intervention with SAC was feasible and effective. Mortality was confined to cases with Hunt and Hess grade V SAH and basilar artery aneurysms—both established predictors of a poor prognosis.^20 21^ The absence of hemorrhagic complications despite early stent placement in the acute SAH setting supports the safety of this approach, particularly when combined with tirofiban bridging and early initiation of prasugrel. The antithrombogenic hydrophilic polymer coating of the pEGASUS-HPC stent may have contributed to the low incidence of thromboembolic events observed, especially in the context of single-catheter techniques and reduced antiplatelet premedication in the acute phase.

### Technical feasibility and device handling

Technical stent deployment was achieved in all cases, underscoring the reliability of the pEGASUS-HPC stent in clinical practice. Moreover, the Y-stent configuration was feasible in all intended cases, indicating sufficient flexibility and radial force for bifurcation coverage. Additionally, the open-cell design and wide struts of the stent facilitated straightforward navigation of the microcatheter through the stent interstices. This feature not only enabled smoother access to the aneurysm sac but also formed the basis for the recently described pusher-assisted catheterization technique where the stent pusher itself is used instead of a microwire to guide the microcatheter into the aneurysm sac.^19^ This technique has been shown to reduce catheterization time significantly—by a factor of seven—while maintaining procedural safety. The wide cells of this feature may contribute to procedural safety by reducing manipulation within the parent artery and minimizing delays during the intervention.

### Comparison with existing literature

Our results compare favorably with previously reported outcomes of other commonly used stent systems for SAC of wide-necked intracranial aneurysms. The Neuroform Atlas, summarized in the meta-analysis by Pranata *et al*,^15^ had an adequate occlusion rate of 88% and a complication rate of 6% across 568 aneurysms whereas our study achieved a higher complete occlusion rate of 92.4% and a substantially lower complication rate of 1.6%. Similarly, in a multicenter analysis of LVIS Jr for unruptured MCA aneurysms, Poncyljusz *et al*^16^ reported a complete occlusion rate of 72.8% at discharge and 81.5% at follow-up, with periprocedural thromboembolic events occurring in 8.6% of cases. In comparison, the pEGASUS-HPC stent yielded more durable occlusion outcomes and fewer thromboembolic events, which may be attributed to its hydrophilic polymer coating. Studies comparing Enterprise and Solitaire stents also showed relevant limitations; although both devices had comparable clinical outcomes, acute in-stent thrombosis was significantly more frequent with the Solitaire stent (12%) and both systems lack antithrombogenic surface modifications.^17^ Taken together, our findings suggest that the use of a modern coated stent such as the pEGASUS-HPC may offer superior safety and efficacy profiles, particularly in complex aneurysm morphologies and in the acute setting.

### Limitations

This study has several limitations. First, its retrospective design introduces the possibility of selection bias and limits causal inference. Second, the absence of centralized image adjudication (eg, core laboratory) may have introduced variability in occlusion grading across centers. Third, procedural and antiplatelet protocols varied between institutions, which could have influenced clinical and angiographic outcomes despite attempts at standardization. Fourth, follow-up imaging was not uniform across centers and included both DSA and non-invasive modalities (MRA, CTA), which may limit the comparability of long-term occlusion assessment. Finally, although our study included Y-stenting cases and ruptured aneurysms, it was not designed to directly compare coated versus uncoated stents.

## Conclusion

SAC using the pEGASUS-HPC stent appears to be a technically feasible and clinically effective treatment option for wide-necked intracranial aneurysms, including those presenting with rupture or requiring Y-stenting. The high rates of immediate and durable occlusion, along with a low incidence of complications, suggest a favorable safety and efficacy profile. The antithrombogenic hydrophilic polymer coating may help reduce the risk of thromboembolism, particularly in the acute setting. While these results are promising, prospective controlled studies are warranted to confirm these observations and further define the role of coated stents in modern neuroendovascular therapy.

## Data Availability

All data relevant to the study are included in the article or uploaded as supplementary information.

## References

[1] Mascitelli JR (2024). Management of wide-neck aneurysms in 2024: how does one make the best treatment decision when there are so many good options?. J Neurointerv Surg.

[2] Molyneux AJ, Kerr RSC, Yu L-M (2005). International subarachnoid aneurysm trial (ISAT) of neurosurgical clipping versus endovascular coiling in 2143 patients with ruptured intracranial aneurysms: a randomised comparison of effects on survival, dependency, seizures, rebleeding, subgroups, and aneurysm occlusion. Lancet.

[3] Darsaut TE, Jack AS, Kerr RS (2013). International Subarachnoid Aneurysm Trial - ISAT part II: study protocol for a randomized controlled trial. Trials.

[4] Belavadi R, Gudigopuram SVR, Raguthu CC (2021). Surgical Clipping Versus Endovascular Coiling in the Management of Intracranial Aneurysms. Cureus.

[5] Haryu S, Sakata H, Matsumoto Y (2024). Endovascular Treatment of Wide-Neck Bifurcation Aneurysm: Recent Trends in Coil Embolization with Adjunctive Technique. J Neuroendovasc Ther.

[6] Campos JK, Lien BV, Wang AS (2020). Advances in endovascular aneurysm management: coiling and adjunctive devices. Stroke Vasc Neurol.

[7] Lee I-H, Ha S-K, Lim D-J (2024). Safety and efficacy of stent-assisted coil embolization with periprocedural dual antiplatelet therapy for the treatment of acutely ruptured intracranial aneurysms. Acta Neurochir (Wien).

[8] Li G, Han Y, Ding S (2022). Stent-assisted coiling of acutely ruptured cerebral aneurysm: a multicenter prospective registry study (SAVE). BMC Neurol.

[9] Tan LA, Johnson AK, Keigher KM (2014). Y-stent-assisted coil embolization of cerebral aneurysms. *FOC*.

[10] Lobsien D, Holtmannspoetter M, Eff F (2024). The pEGASUS-HPC stent system for stent-assisted coiling of cerebral aneurysms: a multicenter case series. J Neurointerv Surg.

[11] Pielenz D, Klisch J, Fiorella D (2025). The pEGASUS-HPC stent system for intracranial arterial stenosis: a single-center case series. J Neurointerv Surg.

[12] Henkes H, Bhogal P, Aguilar Pérez M (2019). Anti-thrombogenic coatings for devices in neurointerventional surgery: Case report and review of the literature. Interv Neuroradiol.

[13] Lenz-Habijan T, Bhogal P, Peters M (2018). Hydrophilic Stent Coating Inhibits Platelet Adhesion on Stent Surfaces: Initial Results In Vitro. Cardiovasc Intervent Radiol.

[14] Boxberg F, Al-Tibi M, Schulz K (2024). Initial Experience with a New Self-Expanding Open-Cell Stent System with Antithrombotic Hydrophilic Polymer Coating (pEGASUS Stent) in the Treatment of Wide-Necked Intracranial Aneurysms. Neurointervention.

[15] Pranata R, Yonas E, Deka H (2020). Stent-Assisted Coiling of Intracranial Aneurysms Using a Nitinol-Based Stent (Neuroform Atlas): A Systematic Review and Meta-analysis. Cardiovasc Intervent Radiol.

[16] Poncyljusz W, Zwarzany Ł, Limanówka B (2020). Stent-Assisted Coiling of Unruptured MCA Aneurysms Using the LVIS Jr. Device: A Multicenter Registry. J Clin Med.

[17] Chen Y-A, Hussain M, Zhang J-Y (2014). Stent-assisted coiling of cerebral aneurysms using the Enterprise and the Solitaire devices. Neurol Res.

[18] Hendricks BK, Yoon JS, Yaeger K (2020). Wide-neck aneurysms: systematic review of the neurosurgical literature with a focus on definition and clinical implications. J Neurosurg.

[19] Alhaj Moustafa B, Khanafer A, Dadak M (2025). pEGASUS HPC stent pusher-assisted catheterization (PAC) of nonruptured cerebral aneurysms: Safety and efficacy. Interv Neuroradiol.

[20] Lenga P, Hohaus C, Hong B (2019). Giant intracranial aneurysms of the posterior circulation and their relation to the brainstem: analysis of risk factors for neurological deficits. J Neurosurg.

[21] Frisoli FA, Srinivasan VM, Catapano JS (2022). Vertebrobasilar dissecting aneurysms: microsurgical management in 42 patients. J Neurosurg.

